# Research experimental design for the construction and identification of the pGEX-BCKD-E4A recombinant point-mutant plasmid

**DOI:** 10.1371/journal.pone.0279431

**Published:** 2023-02-24

**Authors:** Tiannan Zhou, Huixian Wei, Jinjun Wang

**Affiliations:** College of Environmental Science and Engineering, Yangzhou University, Yangzhou, China; University of Hawai’i at Manoa, UNITED STATES

## Abstract

Primary biliary cirrhosis (PBC) is an organ-specific autoimmune disease that eventually develops into cirrhosis and even liver cancer. In recent years, the incidence rate has been increasing, and the early diagnosis and treatment of PBC are crucial. In the early diagnosis method of PBC, anti-mitochondrial antibodies (AMAs) are an important diagnostic basis, especially the M2 subtype (AMA-M2) with almost 100% specificity. We selected the BCOADC-E2 protein, a mitochondrial autoantigen that reacts specifically with AMA-M2 antibodies, and carried out DNA recombination and protein mutation experiments by cloning in vitro the homologous target gene sequence BCKD that expresses the antigenic epitope of BCOADC-E2 protein, to provide experience for later exploring the effect of mutations of amino acids around the lysine in the active center of BCOADC-E2 protein on its specific binding to AMA-M2, and to lay the foundation for determining the key amino acids of BCOADC-E2 for the diagnosis and treatment of PBC. In addition, we apply this scientific research content to graduate course teaching. Experimental technology of microbial molecular ecology is a course with the cross-integration of multidisciplinary knowledge and experimental skills offered at our college since 2018. This article derives from the part of this course on the construction of recombinant plasmids. The students first constructed the recombinant plasmid pGEX-BCKD using the vector plasmid pGEX-4T1 and the target gene fragment BCKD provided by the laboratory and used this as a template to construct the pGEX-BCKD-E4A point mutation plasmid by the overlap extension PCR (SOE PCR) technique to achieve the effect of mutating the fifth amino acid glutamate in front of lysine, the active centre of the BCOADC-E2 lipid acyl binding domain, to alanine for subsequent studies. Through the research experiment, combining theoretical knowledge and experimental operation, we aim to deepen the student’s understanding of DNA recombination technology, let them feel the practical application prospect of experimental technology, stimulate students’ interest in professional knowledge learning, and cultivate students’ scientific thinking and innovation consciousness. We examined the quality of the teaching through the process and summative evaluation of the students. In this study, the students successfully completed the construction of pGEX-BCKD-E4A point mutant plasmid, and the average test score increased from 40.4% before teaching to 91.1%. The teaching effect was remarkable. This kind of research experimental teaching mode has good application prospects, and other education and teachers can refer to and reference it.

## Introduction

Primary biliary cirrhosis (PBC) is an organ-specific autoimmune disease with middle-aged women as the main disease population and eventually develops into cirrhosis and even liver cancer [1], characterized by positive anti-mitochondrial antibody (AMA) [2, 3]. The incidence trend has risen in recent years, but the cause of the disease is still unclear, and smoking, immune disorder and urinary tract infection may induce PBC [4]. Early PBC diagnosis and therapy are very important [5]. In the serum of PBC patients, anti-mitochondrial antibodies (AMAs) are characterized by high titers [6, 7], which can be detected in more than 90% of PBC patients [8, 9]. Therefore, the detection of AMAs is crucial for the diagnosis of PBC [10, 11]. The traditional method for the detection of AMAs mainly relies on immunofluorescence microscopy, which is time-consuming without high specificity [12, 13]. According to the fact that the M2 subtype (AMA-M2) of AMAs antibodies can specifically react with mitochondrial self-antigens with almost 100% specificity [5, 14], PBC can be diagnosed quickly and efficiently by ELISA detection [15–18]. At present, mitochondrial autoantigens known to specifically react with AMA-M2 include pyruvate dehydrogenase complex (PDC-E2), branched-chain 2-oxoacid dehydrogenase complex (BCOADC-E2) and 2-oxoglutarate dehydrogenase complex (OGDC-E2) [12, 19], but mainly react with PDC-E2 [20, 21]. So far, there has been no relevant research on the effect of amino acid mutation near the lysine in the protein’s active center in the fatty acyl-binding domain of BCOADC-E2 protein on its specific binding to AMA-M2 [22, 23]. Therefore, the study is based on the BCOADC-E2 protein to develop relevant experiments.

In the amino acids that constitute proteins, the replacement of certain amino acids with alanine often changes the function of the protein greatly, and we call these amino acids that have a significant impact on the protein function key amino acids. The "Alanine scan" is a mutational technique that replaces an important amino acid residue in a certain target sequence or polypeptide of a protein with a neutral alanine without a special side chain, thus changing the three bases encoding any amino acid to the three bases GCG or GCA or GCC or GCT encoding an alanine. Investigating the changes in the specific properties of the target polypeptide before and after the mutation can identify key amino acid sites that can guide amino acid site-directed mutagenesis [24].

Our experiment was designed to mutate the fifth amino acid glutamate in front of lysine, the active centre of the BCOADC-E2 lipid acyl binding domain, to alanine. BCKD is a homologous target gene sequence cloned from bovine heart tissue capable of expressing a protein containing the BCOADC-E2 lipid acyl binding domain. The recombinant plasmid pGEX-BCKD was constructed by recombining BCKD with the engineered plasmid pGEX-4T1. On this basis, pGEX-BCKD-E4A point mutation plasmids were successfully constructed by SOE PCR [25–28], which provided experience and basis for the later study of effects of amino acid mutation around lysine at the active center of BCOADC-E2 protein on its specific binding to AMA-M2 [29]. This is important for the intensive study of the key amino acids of BCOADC-E2 for the diagnosis and treatment of PBC.

At the same time, based on this basis, we transform the scientific research content into a research-based experimental teaching project, aiming to stimulate students’ interest in learning professional knowledge, help students to master the basic methods of experimental research and cultivate students’ scientific thinking and innovative consciousness [30, 31]. *Experimental technology of microbial molecular ecology* is an elective experimental course intended for first-year postgraduate students of agricultural resources and environment at the college of environmental science and engineering of Yangzhou University in 2018. The theoretical course that goes with it is *molecular biology*. These two courses are offered as elective courses to students interested in molecular biology. Meanwhile, depending on relevant molecular biology experimental technology has become an important way to solve problems in other professional fields. For instance, students who carried out the research of " effects of intestinal microbial communities structure in earthworms on tetracycline degradation" took two elective courses. Before this experiment, there was a theoretical course of *molecular biology* with a total of 12 class hours (about 2 h per class hour), and 2 class hours of teaching conducted every week. The theoretical knowledge involved in the experiment was especially explained. To let students know more about the cutting-edge progress of molecular biology, during this period, Professor Liang from the University of California, Davis was specially invited to give a total of four class hours of teaching entirely in English for everyone, with good results achieved. At present, what is carried out is the construction and mutation experiment of recombinant plasmids related to DNA recombination technology—the construction of pGEX-BCKD recombinant plasmids, which is taken as a template to construct pGEX-BCKD-E4A point mutation plasmids by gene splicing by overlap extension PCR (SOE PCR) technology for follow-up research.

### Experimental design

The whole experiment can be summarized into the following six parts (Table 1), and each part is guided by a special teacher or experiment instructor. Each postgraduate student needs complete the experiment independently. In this experiment, although the theoretical knowledge has been introduced in detail in the course of *molecular biology*, the key points of operation in the experiment, especially the use and protection of some dangerous reagents, such as EB, should be emphasized again.

**Table 1:** Planning and arrangement of the experimental course.

Serial number	Task	Class hour	The goal of the experiment
1	Construction of recombinant plasmid pGEX-BCKD	1	Completion of the correct construction of the recombinant plasmid
2	Identification of recombinant plasmid pGEX-BCKD	1	Master the detection method for agarose gel electrophoresis
3	Design of mutation primers	1	Learn to design positive and negative specific mutation complementary primers with mutated genes
4	SOEPCR site-directed mutagenesis of pGEX-BCKD recombinant plasmids	1	Master SOE PCR technology
5	enzymolysis and transformation of amplified products	1	Removal of non- target cloned products by enzymolysis to complete extended culture
6	Extraction of pGEX-BCKD-E4A point mutation plasmids	1	Extraction and completion of the extended culture of monoclonal colonies

## Materials and methods

### Participants

Our participants are ten postgraduates in our college, and we obtained all of their consent verbally to participate in this experiment. Our study is exempt from the need for approval and confirmation from the institutional review board. Because the experiment is not put in clinical human or animal experiments, there is no ethical risk. Also, the experiment is a part of the course of Experimental Technology of Microbial Molecular Ecology opened by our university since 2018 and the experimental content and mode fully meet the standards of the Ministry of Education in our nation. Therefore, the students were obliged and fully agreed to perform this experiment. The data that we give for the evaluation of teaching outcomes are completely anonymous with no personal information being collected. The data is not considered to be sensitive or confidential in nature. And the issues being researched are not likely to upset or disturb participants. Vulnerable (older than 59 years old and younger than 19 years old) or dependent groups are not included.

### Materials and instruments

#### Materials

BamHI restriction endonuclease, T4 DNA ligase, 2×TransStart Fast Fly SuperMix, DMT enzyme and DMT-competent cells were all provided by TransGen Biotech Co., Ltd; Ampicillin (Amp), DNA marker 2000, SanPrep column plasmid DNA Mini-Preps extraction kit and primer synthesis were all provided by Sangon Biotech (Shanghai) Co.,Ltd.; the target gene BCKD and plasmid vector pGEX-4T1 were provided by University of California, Davis; ddH2O and other materials are prepared by the lab of environmental science and engineering, Yangzhou University.

#### Instruments

Bio-Rad T100 gradient PCR instrument, JY600 electrophoresis apparatus, gel imager, ultra-pure water machine, circulating water bath, ultra-high speed centrifuge, constant temperature shaking table, etc.

### Methods

#### Construction of recombinant plasmid pGEX- BCKD

**Fig 1:**
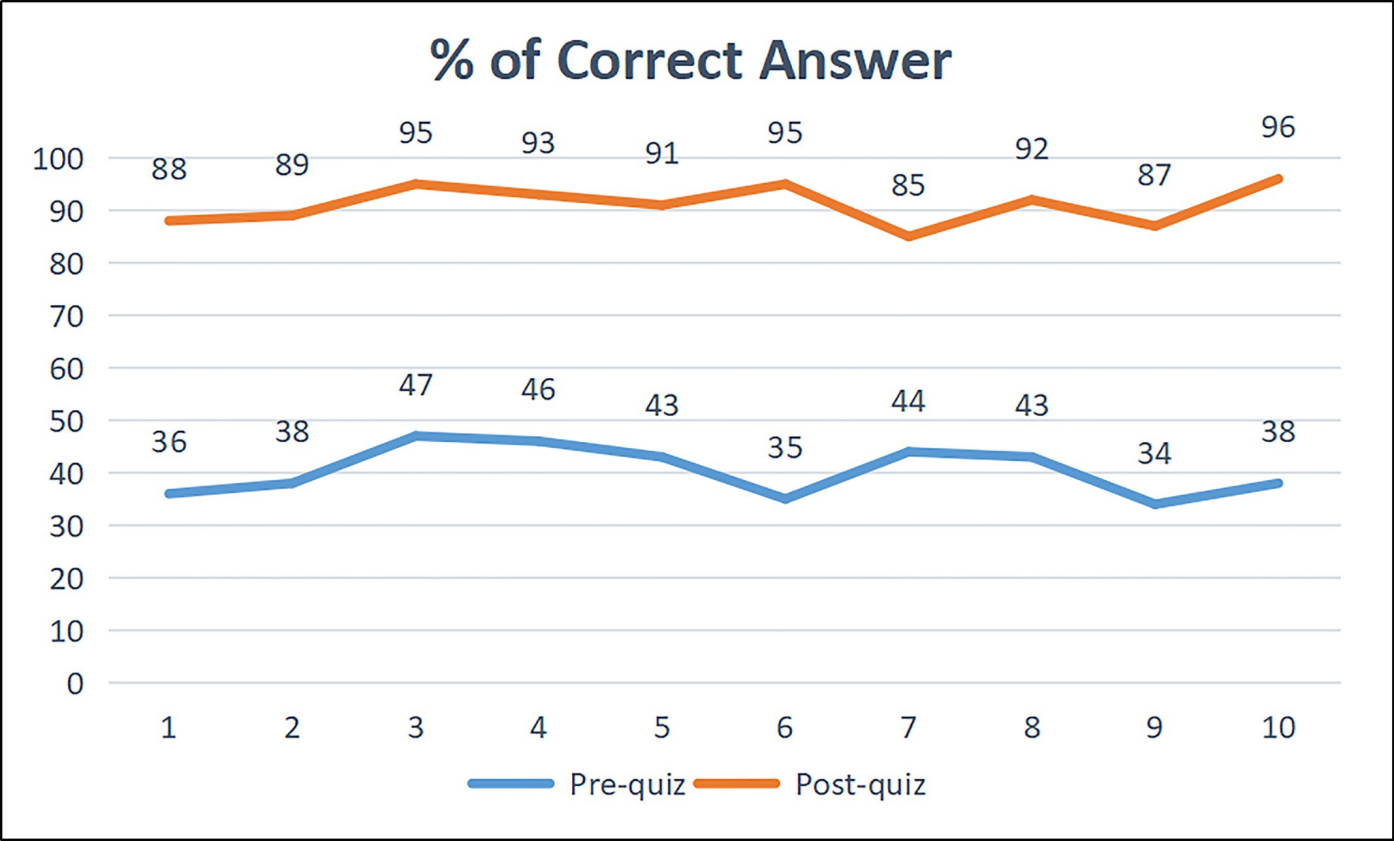
The construction of pGEX-BCKD recombinant plasmid.

Enzyme digestion was performed on the target gene BCKD and plasmid vector pGEX-4T1 with BamHI restriction endonuclease to make them have the same GATC sticky ends. Then, T4 DNA ligase was added to connect the sticky ends of target gene BCKD and plasmid vector pGEX-4T1 by complementary pairing and the recombinant plasmid was named pGEX-BCKD. Next, the schematic diagram of the construction of the pGEX-BCKD recombinant plasmid is shown in Fig 1, which can allow students to understand the whole recombination process more intuitively.

In this part of the teaching experiment, students should be asked to compare the advantages and disadvantages of single enzyme digestion and double enzyme digestion systems, complete Table 2 and think about the reasons for choosing a single enzyme digestion system in this experiment, to deepen their understanding.

**Table 2:** Comparison between the two digestion methods.

Type	Single enzyme digestion	Double enzyme digestion
Advantages		
Disadvantages		

The students were instructed to add 5 μL recombinant plasmid product to the freshly thawed DH5a competent cells, followed by an ice bath for 30 min after flicking and mixing; the centrifuge tube was placed in a 42°C water bath kettle for 45s and immediately put on the ice for 2min, during which the centrifuge tube was kept stable. Then, 250 μL of LB (or SOC) medium (without ampicillin) pre-heated to 37°C was added to the centrifuge tube, mixed evenly and placed in a shaking table at 37°C, 200 rpm/min for culture for 1h to make the resistance gene on the plasmid express and the competent cells resuscitate. After resuscitation, the bacteria were evenly coated on the LB plate containing Amp resistance and the plate was inverted and cultured in a 37°C incubator for 14h. Next, the growth of the colonies on the plate was observed until a single colony appeared, and then the single colony was picked for extended culture. Finally, the pGEX- BCKD recombinant plasmid was extracted according to the instructions of the SanPrep Column Plasmid Mini-Preps Kit.

In this part of the experiment, during instructions for students, there are the following points to pay attention to:

① The amount of bacterial solution used to coat the plate should be moderate. Too much bacterial solution is not easy to form monoclonal colonies while too little bacterial solution is easy to cause no colony growth.
② The heat shock time should be strictly controlled. Too long heat shock time is easy to cause the death of competent bacteria while too short heat shock time is easy to cause low transformation efficiency.
③ After 14h, the growth of colonies on the plate should be closely observed, and the maximum culture time should not exceed 16h. With the growth of monoclonal colonies containing ampicillin resistance, ampicillin near the monoclonal colonies can be decomposed, thereby resulting in satellite colonies around the monoclonal colonies. The appearance of satellite colonies indicates that the culture time is too long.
④ As many single colonies as possible should be picked for inoculation and extended culture to screen out the false-positive single colonies caused by the cyclization of the plasmid vector itself.
⑤ The extended culture time should not exceed 16h, and the over-cultured plasmid will be degraded.

#### Identification of recombinant plasmid pGEX-BCKD

The theoretical size of the recombinant plasmid pGEX-BCKD obtained by the students should be 5329bp, and thus the size of extracted recombinant plasmids was detected by 1% agarose gel electrophoresis. 10 μ L plasmid DNA samples and 2 μ L 6 × DNA loading buffer were taken and mixed evenly and 10 μ L mixture was pipetted out for sample application and 3 μL DNA standard molecular weight (DNA Marker) was also pipetted for sample loading. The electrophoresis voltage and the time were set to 115V and 25min respectively. Meanwhile, the BamHI restriction enzyme was used for Enzyme digestion of the recombinant plasmid, and the size of the enzyme-digested product was verified by 1.5% agarose gel electrophoresis. The interference of false positive single colonies caused by self-cyclization of plasmid vector pGEX-4T1 was excluded. After each student completed the preliminary verification, the plasmid was sent to Sangon Biotech (Shanghai) Co., Ltd. for sequencing. The sequencing primer was the pGEX 5’-end sequencing primer at 61 bp upstream of the enzyme digestion site: GGGCTGGCAAGCCACGTTTGGTG.

In this part of the experiment, during instructions for students, there are the following points to pay attention to:

① The experimental instructor needs to explain to students the effect of the concentration of agarose gel on the separation of DNA with different molecular weights, and also elucidate the principle of electrophoresis to guide students to analyze the electrophoresis results by themselves.
② When students use ethidium bromide (EB) to make agarose gel, it is essential that an experiment instructor should be arranged to constantly observe the operation of students to ensure the safety of the experiment.

#### Design of mutation primers

According to the gene sequence of pGEX-BCKD recombinant plasmids, students were guided to design two forward and reverse mutation-specific complementary primers with mutated genes at the position where the target gene fragment of BCKD needs to be mutated. The primers were all 43bp in length to ensure the specific binding of mutation primers to the plasmid template, and also reduce the generation of nonspecific products in the process of SOE PCR. The mutant primer sequence is shown in Table 3.

**Table 3:** The mutation primer sequence.

Primer	Sequence
MutagenicForwardprimer	5’-ctcagtttgatagcatctgtg **c** agttcaaagtgataaagcttc-3’
MutagenicReverseprimer	5’-gaagctttatcactttgaact **g** cacagatgctatcaaactgag-3’

#### pGEX-BCKD recombinant plasmids of SOEPCR site-directed mutation

The instructor first strengthened the experimental principle for the students: the original plasmid was taken as the template, and two specific mutation primers are complementally paired with the original plasmid(excluding mutation sites). Both ends of the primer are extended by pfu high-fidelity enzyme, and finally, form two closed circular DNA strands containing mutated genes. The complementary pairing of two mutant DNA strands forms the point mutation plasmid pGEX-BCKD-E4A. Then, taking the pGEX-BCKD recombinant plasmid constructed by the laboratory in the early stage as the cloning template, the students used SOE PCR technology for site-directed mutagenesis of the recombinant plasmid pGEX-BCKD and the mutated product was named pGEX-BCKD-E4A. The SOE PCR reaction system: 25 μL of 2 × TransStart Fast Fly SuperMix, 1μL forward mutation primer (10 μ M), 1 μ L reverse mutation primer (10 μ M), 1 μL recombinant plasmid template (13.2ng/ μ L), 22 μL nuclease-free water. SOE PCR reaction conditions were as follows: pre-denatured at 94°C for 5 min, denatured at 94°C for the 20s, annealed at 60°C for 20s and extended at 72°C for 2min and this process was repeated at 25 cycles; extended at 72°C for 10min and stored at 4°C. Finally, 10 μL cloned product was taken and detected by agarose gel electrophoresis with a mass concentration of 1%.

#### Enzymolysis and transformation of amplification products

SOE PCR cloned products consist of a small amount of pGEX-BCKD recombinant plasmids and the target cloned product pGEX-BCKD-E4A point mutation plasmids. Therefore, the addition of the DMT enzyme enables specific Enzymolysis of pGEX-BCKD recombinant plasmids to isolate pGEX-BCKD-E4A point mutation plasmids. Students needed to thoroughly remove the recombinant plasmid pGEX-BCKD under the specified Enzymolysis system and conditions, and gently flick and evenly mix 20 μ LSOE PCR product, 1 μ L DMT enzyme and 9 μ LddH2O, followed by enzymolysis at 37°C for 2.5h. After enzymolysis, the centrifuge tube containing 50 μ L competent cells was placed on the ice for thawing, and 5 μ L enzymolysis product was added when the competent cells were just thawed, gently flicked, evenly mixed and followed by an ice bath for 30min; the centrifuge tube was placed in a 42°C water bath for 45s and immediately placed on the ice for 2min, with no shaking the centrifuge tube during this process. Next, 250 μL SOC medium pre-heated to 37°C was added to the centrifuge tube, and mixed evenly; the centrifuge tube was placed in a shaker at 37°C, 200rpm / min and cultured for 1h to resuscitate the bacteria. The LB solid culture containing ampicillin (AMP) was preheated in a 37°C incubator for 30min, and 100 μL the resuscitated bacterial solution was pipetted out and coated evenly on the LB plate; after inversion of the plate for culture at 37°C for 14 h, the colony growth on the plate was observed.

#### Extraction of pGEX-BCKD-E4A point mutation plasmids

Students should randomly pick out monoclonal colonies on the plate and inoculate them into a 5ml LB liquid medium containing ampicillin for extended culture. During this process, the instructor needs to observe whether the students’ operation was standardized to prevent bacterial contamination. The plasmid was extracted according to the instructions of the SanPrep Column Plasmid Mini-Preps Kit and sent to Sangon Biotech (Shanghai) Co., Ltd. for sequencing. The sequencing primer was PGEX5’end sequence: GGGCTGGCAAGCCACGTTTGGTG.

This experiment involved multiple times of sample delivery and sequencing. It was necessary to instruct students to correctly fill in the sample order, including plasmid sample number, sequencing primers, length of the tested fragment, plasmid resistance, purification methods, etc.

## Results

### Identification results of recombinant plasmid pGEX-BCKD

**Fig 2:**
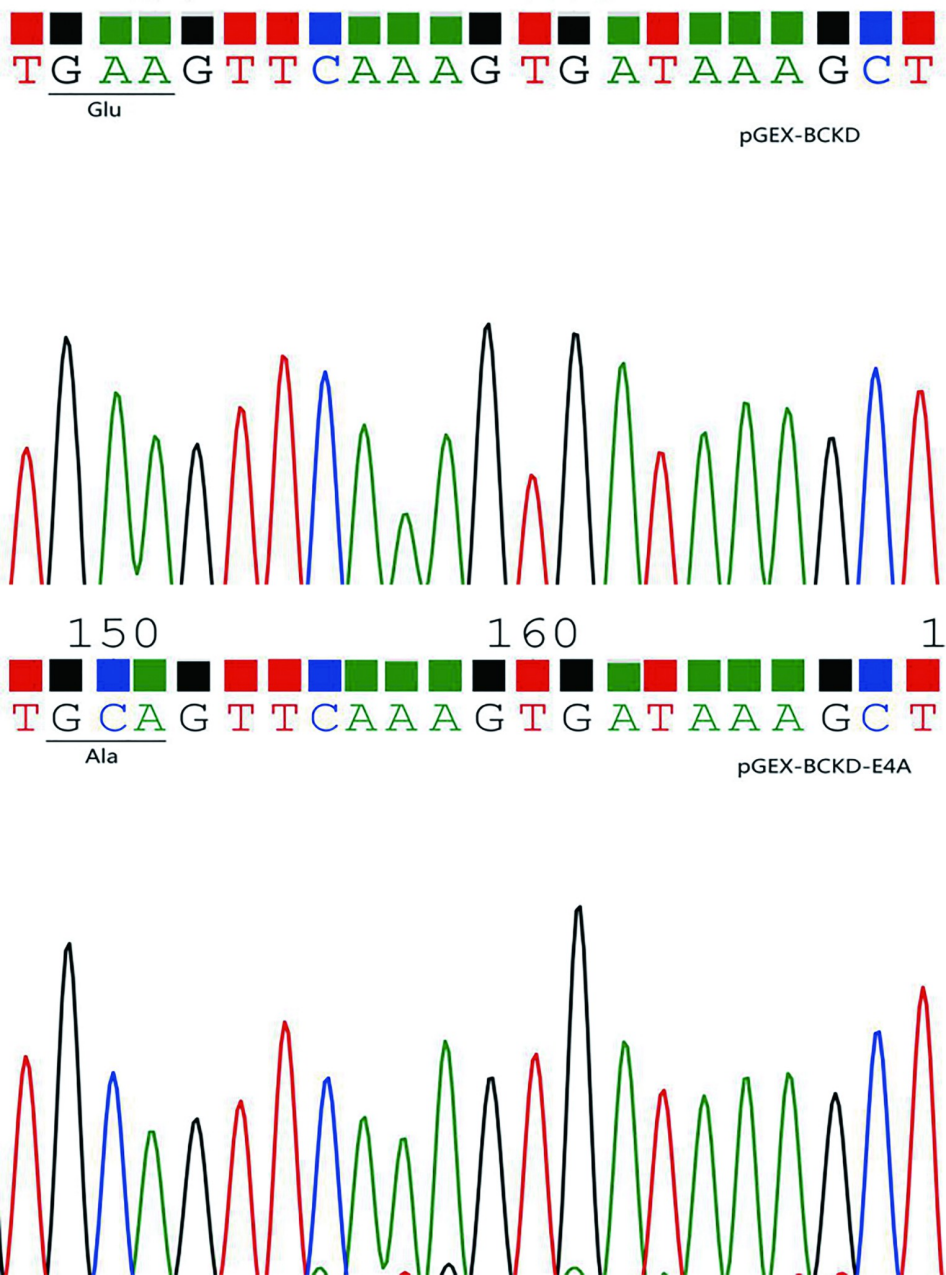
Agarose gel electrophoresis of pGEX-BCKD.

**Fig 3:**
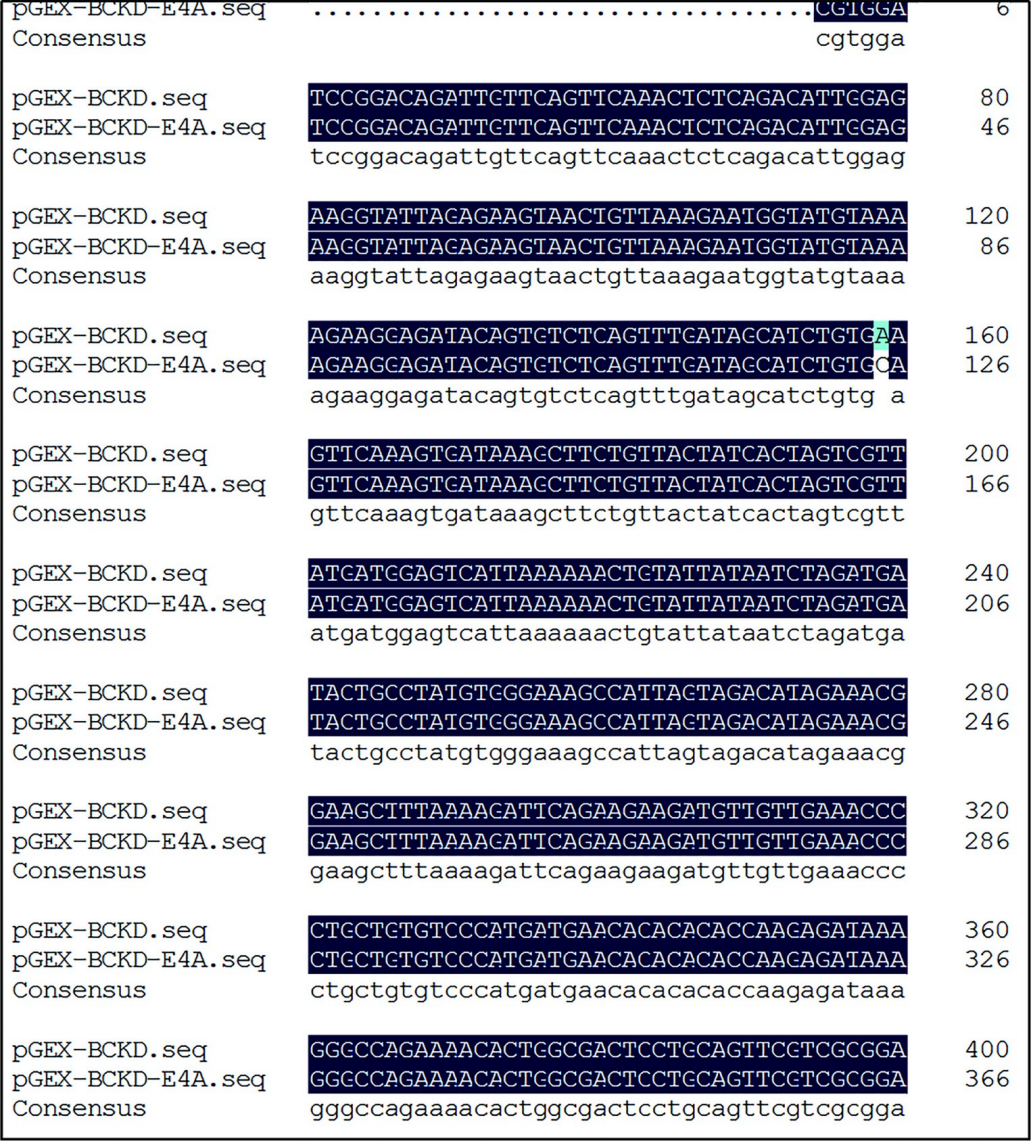
Agarose gel electrophoresis of pGEX-BCKD enzyme digestion.

**Fig 4:**
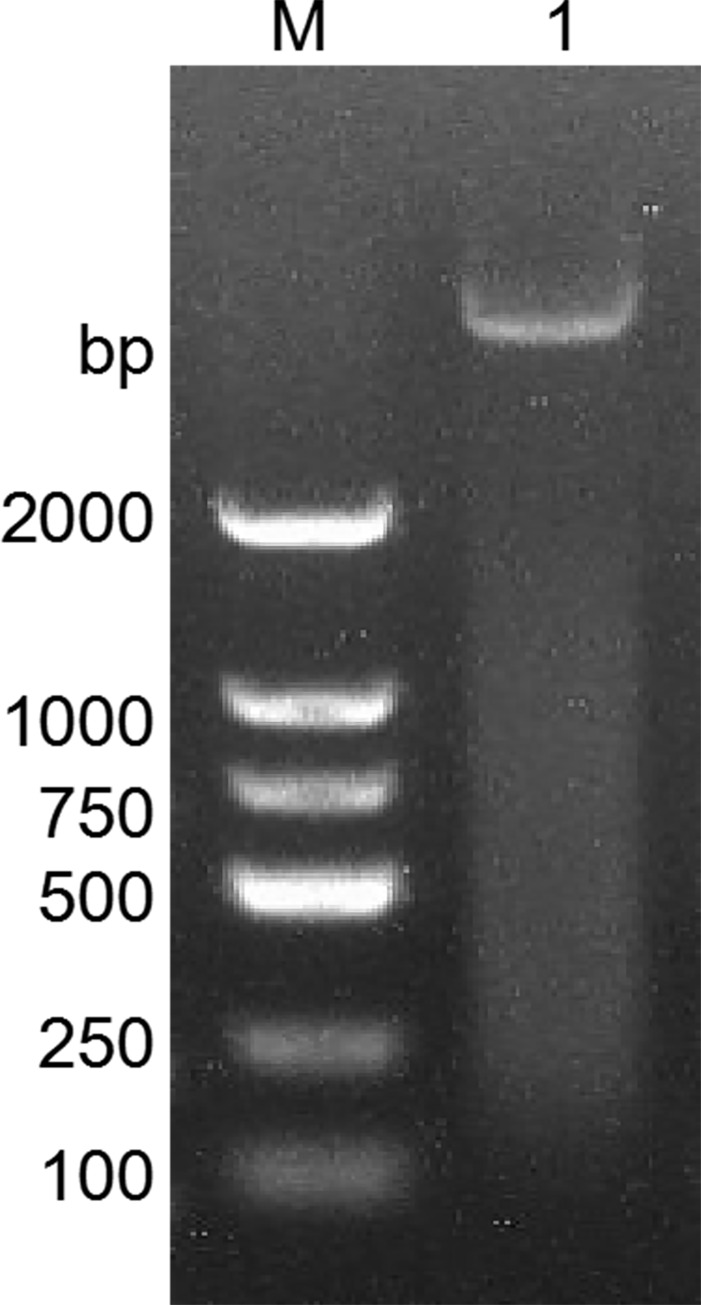
Blast result of pGEX-4T1 and pGEX-BCKD.

The results of the detection of recombinant plasmid pGEX-BCKD by 1% agarose gel electrophoresis completed by students are shown in Fig 2. The electropherogram showed that a band of about 5000bp is clearly visible above the 2000bp DNA marker, and it is consistent with the pGEX-BCKD recombinant plasmid with a theoretical length of 5369bp, which preliminarily indicated the successful construction of the pGEX-BCKD recombinant plasmid. As shown in Fig 3, enzyme-digested products of pGEX-BCKD recombinant plasmids were subjected to 1.5% agarose gel electrophoresis. The electrophoresis bands showed that there were two clear electrophoresis bands, and they were located at about 5000bp and within 250bp-500bp respectively, which are consistent with the theoretical gene fragments of 4969bp and 360bp, indicating the existence of exogenous gene fragments successfully fused with the pGEX-4T1 plasmid vector. Then, the students compared the sequencing results of pGEX-BCKD recombinant plasmids with the plasmid vector pGEX-4T1. The comparison results are shown in Fig 4. The results showed that the insertion site of the target gene BCKD was correct, and so was the gene sequence, indicating the successful construction of the pGEX-BCKD recombinant plasmid.

### Identification of SOEPCR cloned products

**Fig 5:**
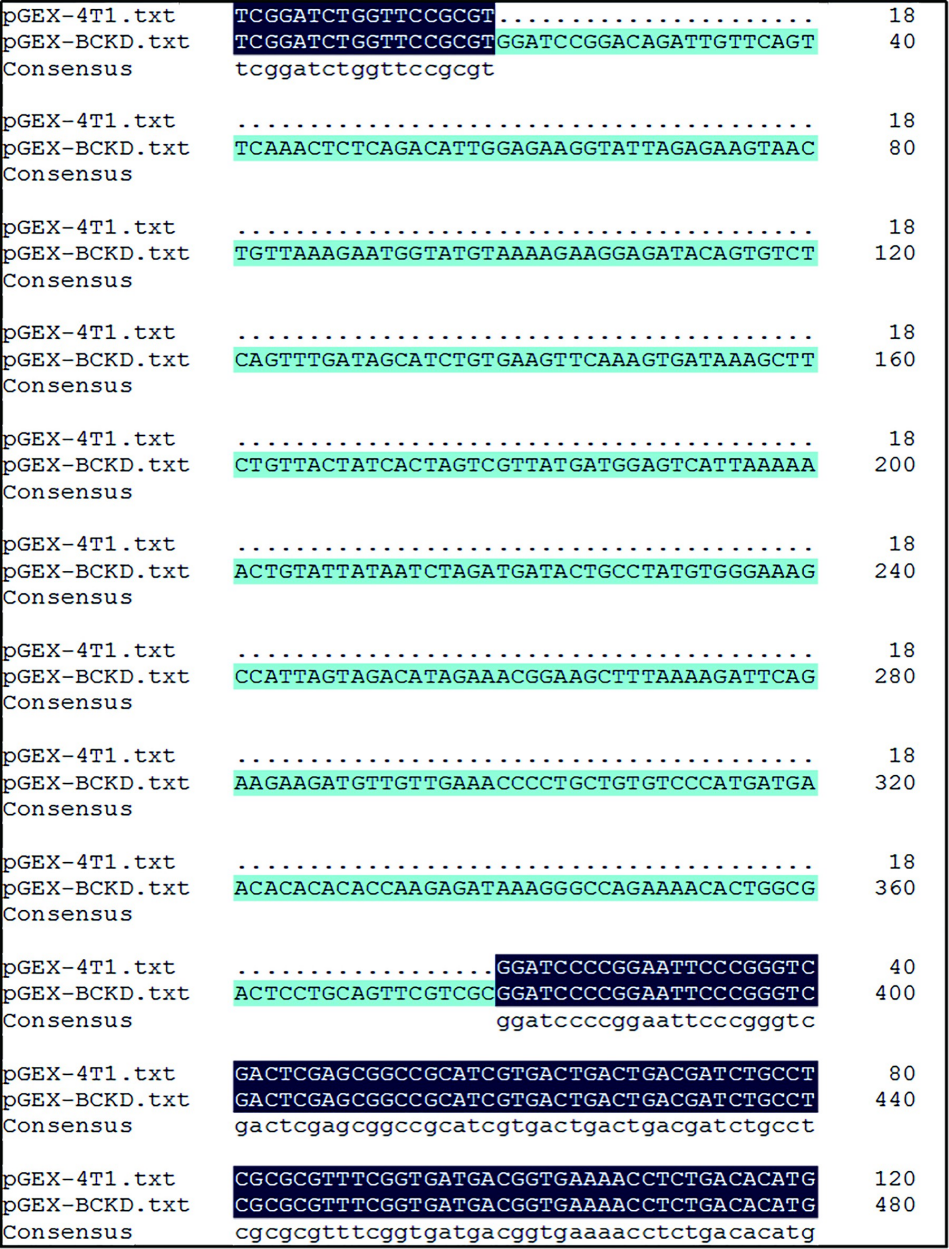
Agarose gel electrophoresis of colony SOE PCR.

The results of the detection of SOE PCR cloned products by 1% agarose gel electrophoresis are shown in Fig 5 the target cloned band exists at about 5000bp, and the size was correct, indicating that the size of SOE PCR cloned products obtained by the students using the pGEX-BCKD recombinant plasmid as a template is in line with the expectations. However, it requires subsequent sequencing whether the gene at the designated position is mutated correctly.

### Sequencing results and analysis of point mutation plasmid pGEX-BCKD-E4A

**Fig 6:**
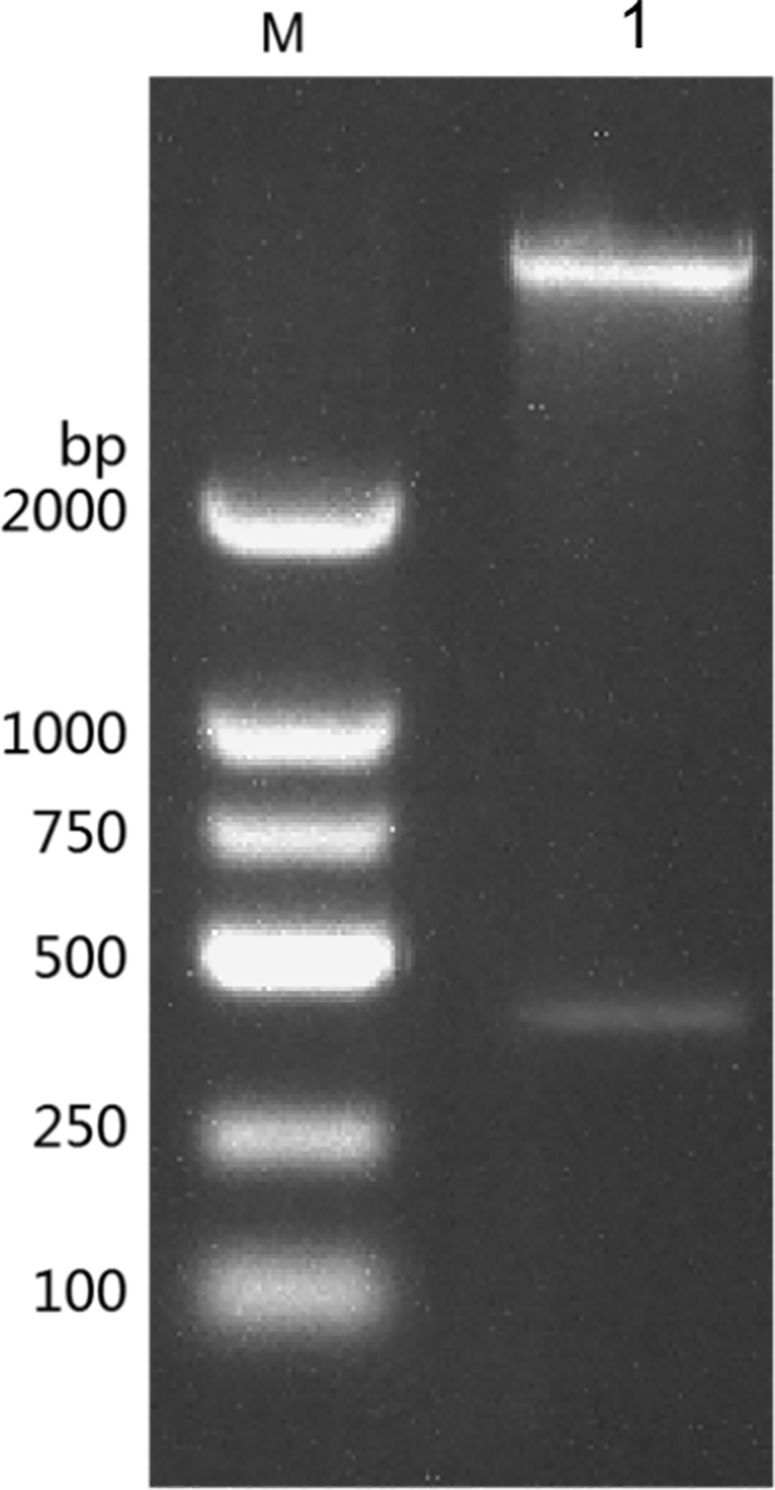
The sequence map of pGEX-BCKD and pGEX-BCKD-E4A.

**Fig 7:**
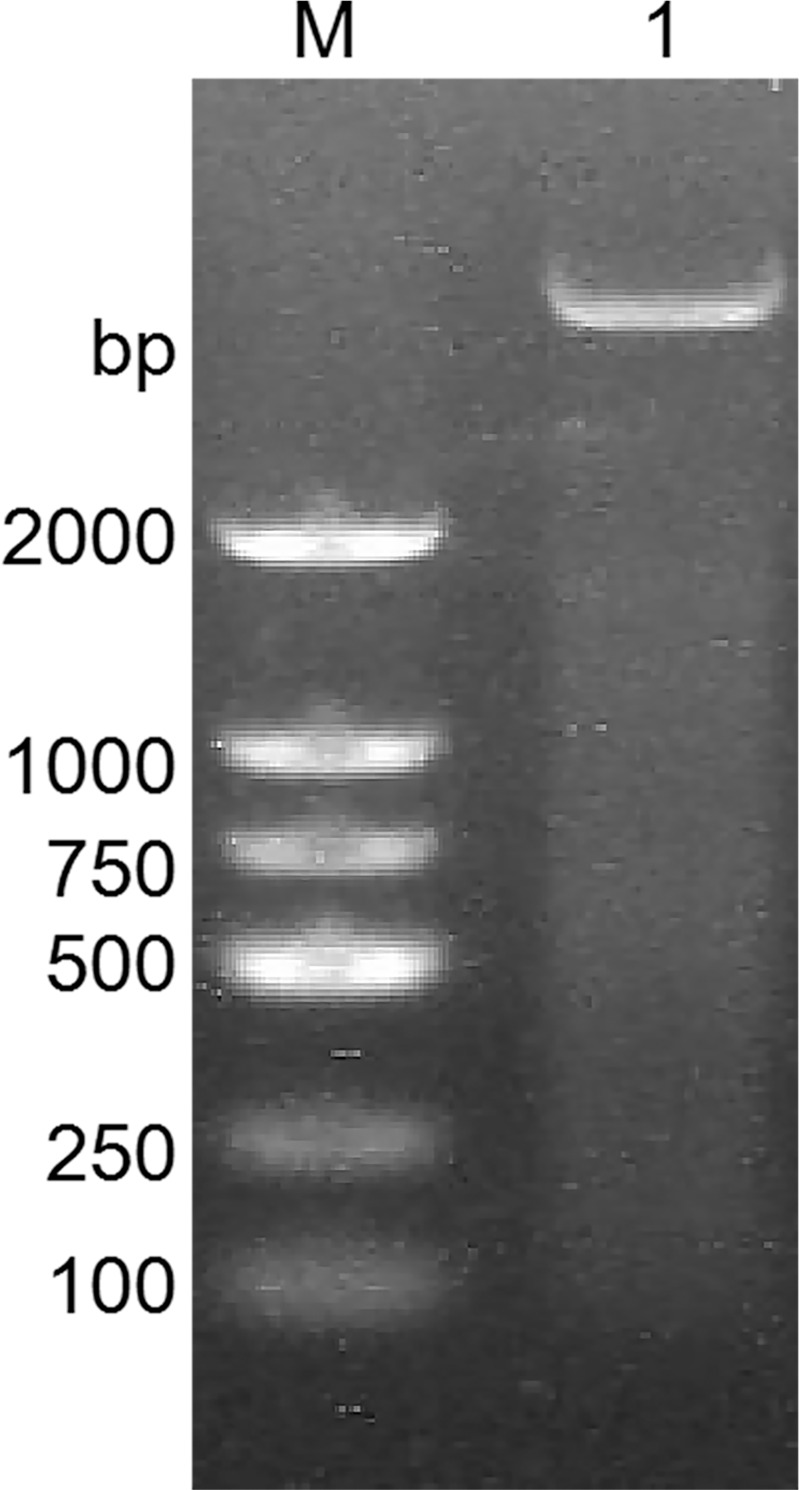
The sequencing results of pGEX-BCKD and pGEX-BCKD-E4A.

The gene sequencing results of SOE PCR cloned products after enzymolysis, transformation and plasmid extraction were compared with pGEX-BCKD gene sequences, as shown in Fig 6. By the comparison, the following findings were obtained: at the mutation site the original three-base GAA encoding glutamic acid (Glu) was mutated to three-base GCA encoding alanine (Ala), and the remaining sequences were completely consistent with the BCKD sequence; the peaks and troughs of the sequencing profile were clear and there was no interference of miscellaneous peaks, indicating that the sequencing results are reliable, as shown in Fig 7. Only after the above-mentioned series of identification and comparison can it be confirmed that the students’ pGEX-BCKD-E4A point mutation plasmids were successfully constructed.

## Discussion

### Analysis of experimental results

At present, the detection of AMAs in human serum is still a very important method for the diagnosis of PBC [32–35]. However, most current studies target PDC-E2 in mitochondrial autoantigens, and few on BCOADC-E2 [36–38]. The effect of the amino acid mutation near the lysine in the active center of the BCOADC-E2 protein on its specific binding to AMA-M2 is completely in the research gap. This experiment was aimed at the specific antigen-antibody reaction between AMA-M2 and BCOADC-E2 to obtain the homologous target gene BCKD that can express the BCOADC-E2 protein. Firstly, the recombinant plasmid pGEX-BCKD that can express BCOADC-E2 protein in vitro was constructed, on which basis the point mutation plasmid pGEX-BCKD-E4A was successfully constructed. The successful construction of recombinant plasmid pGEX-BCKD greatly facilitated the mutation of target gene BCKD at different mutation sites and the mutation plasmid could be obtained only by one PCR amplification in the whole mutation process, with a short time spent and a high mutation success rate (> 93%), which resulted in a higher success rate for the experiments of students, the facilitation of experimental teaching and the stimulation of their research interest. Meanwhile, the successful construction of pGEX-BCKD-E4A point mutation plasmids provides experience for the primer design, the system and conditions of SOEPCR amplification, the usage amount of PCR products and enzymolysis time during enzymolysis, the usage amount of transformation of enzymolysis products and the usage amount of bacterial solution for coating, etc. in the subsequent amino acid mutation experiment, which is conducive to the expansion of follow-up experimental research teaching. The "alanine scanning" of the fatty acyl binding domain of BCOADC-E2 protein through subsequent experiments laid a foundation for studying the effect of amino acids around the active center of BCOADC-E2 protein on its overall protein functional expression [39], and in turn, screening the amino acids that have a key impact on the specific binding of BCOADC-E2 protein to AMA-M2.

### The evaluation of experimental effectiveness

#### Reports and tests

At the beginning of the experimental course, each student received an experimental report to record their experimental situation at each stage. The contents to be filled in the report include the experimental title, the experimental goal, experimental instruments and reagents, experimental operation steps, data records and results, feelings and experiences, and existing questions. At the end of the course, the effectiveness of the experiment was preliminarily evaluated according to the students’ experimental report.

**Fig 8:**
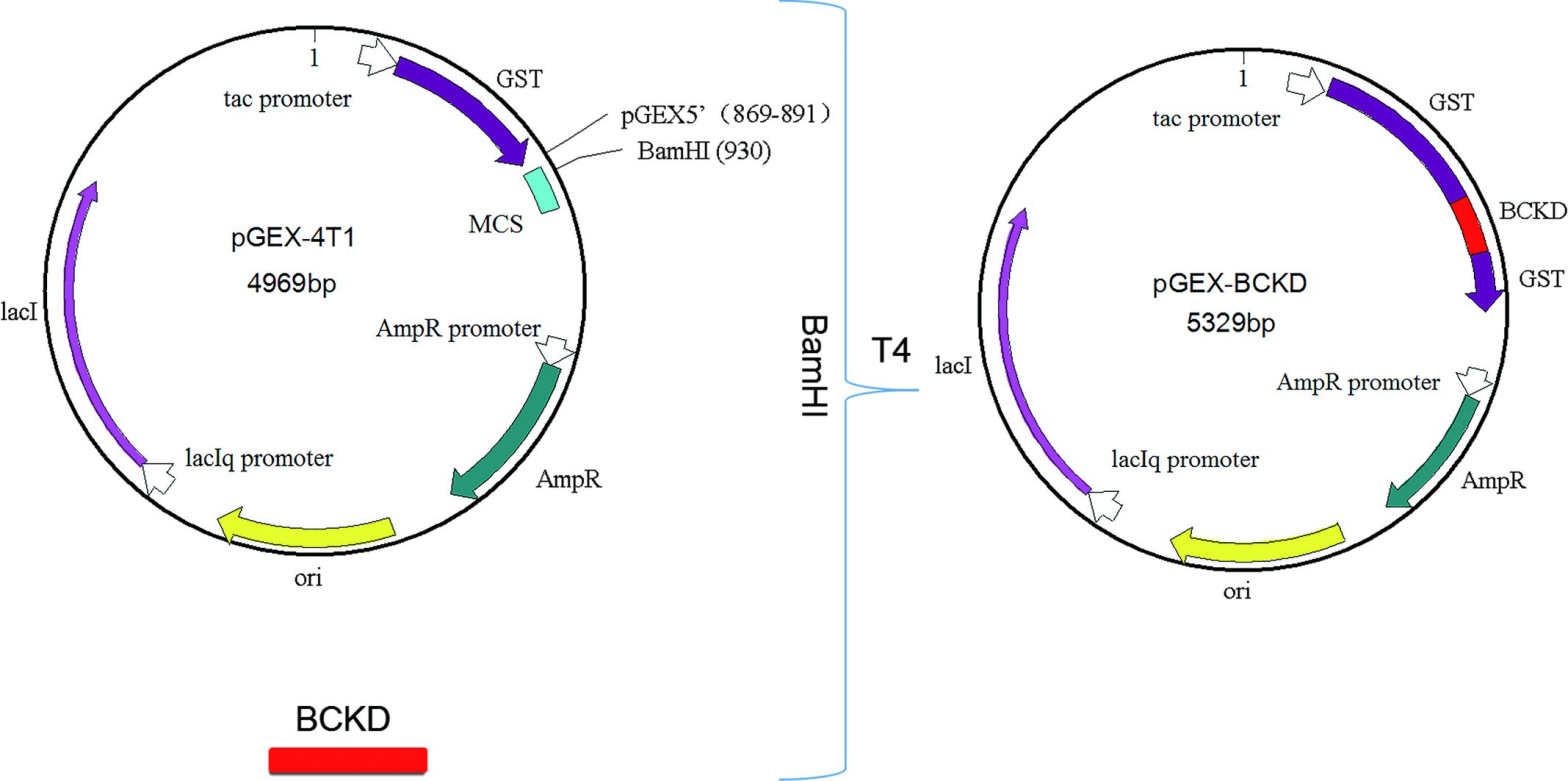
Correct accuracy of 10 students in the front and back tests. Each data represents the total accuracy rate when the student completed the test.

Additionally, in the experimental course, the final examination and evaluation were also performed on the students according to the test scores. By comparison of the pre-test scores in the early stage of the course and the post-test scores after the course learning, it was found that the ability of the students has been significantly improved. After the scores of 10 students in the research group were compared and made statistics, the results obtained are shown in the following Table 4 and Fig 8.

**Table 4:** Comparison of the test average performance results.

	Pre-quiz	Post-quiz
Average%	40.4%	91.1%

### Test questions attached: (Correct answers are bolded)

1. The basic construction process of recombinant DNA is to (C)

A. splice any two segments of DNA together

B. insert exogenous DNA into human DNA

**C. insert the target gene into an appropriate vector**

D. insert the target gene into mammalian DNA

E. insert exogenous DNA into host genes

2. DNA cleavage of restriction endonuclease generates (B)

A. 3-phosphate terminal and 5-hydroxy terminal

**B. 5-phosphate terminal and 3-hydroxy terminal**

C. 3-phosphate terminal and 5-phosphate terminal

D. 5-hydroxy terminal and 3-hydroxy terminal

E. 3-hydroxy terminal, 5-hydroxy terminal and phosphoric acid

The same plasmid DNA exists in three different forms. and their migration rates during electrophoresis are (A)

**A.SC DNA > L DNA > OC DNA**

B.OC DNA > SC DNA > L DNA

C.L DNA > OC DNA > SC DNA

D.SC DNA > OC DNA > L DNA

4. During the identification of recombinant plasmid pGEX-BCKD, in addition to 1% agarose gel electrophoresis detection, 1.5% agarose gel electrophoresis detection need also be completed what is it for? (D)

A. Reducing experimental error

B. Serving as a control group

C. Exclusion of the effect of BCKD self cyclization

**D. Exclusion of the effect of self-cyclization of plasmid vector pGEX-4T1**

5. Which of the following is true about recombinant DNA technology (ABCD)

**A. Recombinant DNA molecules can enter host cells by transformation or transfection**

**B. Recombinant DNA consists of vectors and target DNA**

**C. Restriction endonuclease is one of the tool enzymes**

**D. Plasmids and phages can both be used as vectors**

6. Influence factors of DNA mobility in electric field (ABCDE)

**A. Molecular size, shape and conformation of DNA**

**B. Agarose concentration**

**C. Applied voltage**

**D. Embedded dye**

**E. Composition of electrophoresis buffer**

7. Under what circumstances can satellite colonies appear on the transformation plate and why do satellite colonies appear?

**Ans:** the culture time of the coated medium is too long. The accumulation of expression products of resistance genes on the vector due to the long culture time results in the ineffectiveness of the antibiotics around monoclonal colonies containing ampicillin resistance, which in turn makes the empty colonies without plasmids grow in large quantities, thereby leading to the appearance of satellite colonies.

8. The application of SOE PCR in such aspects as gene site-directed mutagenesis and target gene amplification is extensive and unique. How are its primer design principles different from those of ordinary PCR primers? What aspects should be paid attention to in the design of SOEPCR primers?

#### Ans

① There is no need to design enzyme digestion recognition sites to connect DNA fragments from different sources

② Gene splicing does not require adaptor processing and ligase but utilizes PCR.

③ The conditions are simple, and there is no need to synthesize genes or find templates for the acquisition of a large number of target gene copies.

④ The yield of target products is highly specific and no construction of a library is required for screening.

⑤ High fidelity point-directed and site-directed mutagenesis can be performed, and mutation and recombination can be performed simultaneously.

⑥ Due to the large conditional inertia of the reaction, random mutations are prone to occur, and thus PCR conditions need to be optimized.

### Homework and exploration

Students need to continue to explore the subsequent expression of recombinant point mutation plasmid pGEX-BCKD-E4A and think about the factors that affect the expression of the required BCOADC-E2 protein and its function after expression and its expression level after class. Their personal opinions also need to be completed and submitted within one week after the course ends. The assignment is intended to improve students’ independent thinking and investigation ability, which is also a very important skill to develop at the postgraduate level.

## Conclusion

To sum up, we asked students to practice the operation of core biotechnology for a limited time. Given that this experiment was characterized by less content, relatively few experimenters and relatively flexible experiment time, everyone was required to complete the experiment independently. Of course, the students could communicate with each other during the experiment or after class. Everyone was expected to participate in every step of the application of plasmid recombination technology. Ultimately, all 10 students successfully constructed the point-mutant plasmid, pGEX-BCKD-E4A. In the experiment, students learned to use a variety of technologies, such as the construction of recombinant DNA molecules, the introduction of recombinant DNA molecules into recipient cells, the screening of monoclonal colonies and the culture of microorganisms, plasmid extraction, and electrophoresis. Through experimental guidance, their experimental operation skills in molecular biology and microbiology have been improved. Also, the students personally participated in the construction of recombinant plasmids, which greatly satisfied their curiosity about genetic engineering. In addition, the experiment also involved a lot of microbiology-related knowledge and experimental skills, and the cross-integration of multi-disciplinary knowledge and experimental skills could greatly improve the comprehensive quality of students.

The design of this research experiment was generally successful and we believe that this model of transforming scientific research content into a research experiment teaching project has good prospects for application, especially for the direction of biochemistry education, which has high requirements for mastering cutting-edge experimental techniques. Our teaching model is therefore just right for laying the foundations for subsequent research, while allowing students to get close to the frontiers of science and carry out practice personally, rather than just staying with the theoretical knowledge in textbooks. This is a model that other educators can refer to and learn from.

## Supporting information

S1 Raw images Fig 2. Agarose gel electrophoresis of pGEX-BCKD. Fig 3. Agarose gel electrophoresis of pGEX-BCKD enzyme digestion. Fig 5. Agarose gel electrophoresis of colony SOE PCR.
(PDF)
