# Supplementary material for: Research experimental design for the construction and identification of the pGEX-BCKD-E4A recombinant point-mutant plasmid
Source: PLoS One. 2023 Feb 24;18(2):e0279431. doi: 10.1371/journal.pone.0279431 (PMC9955595; doi:10.1371/journal.pone.0279431)
Supplement: S1 Raw images — Fig 2. Agarose gel electrophoresis of pGEX-BCKD. Fig 3. Agarose gel electrophoresis of pGEX-BCKD enzyme digestion. Fig 5. Agarose gel electrophoresis of colony SOE PCR. (PDF) [file pone.0279431.s001.pdf]

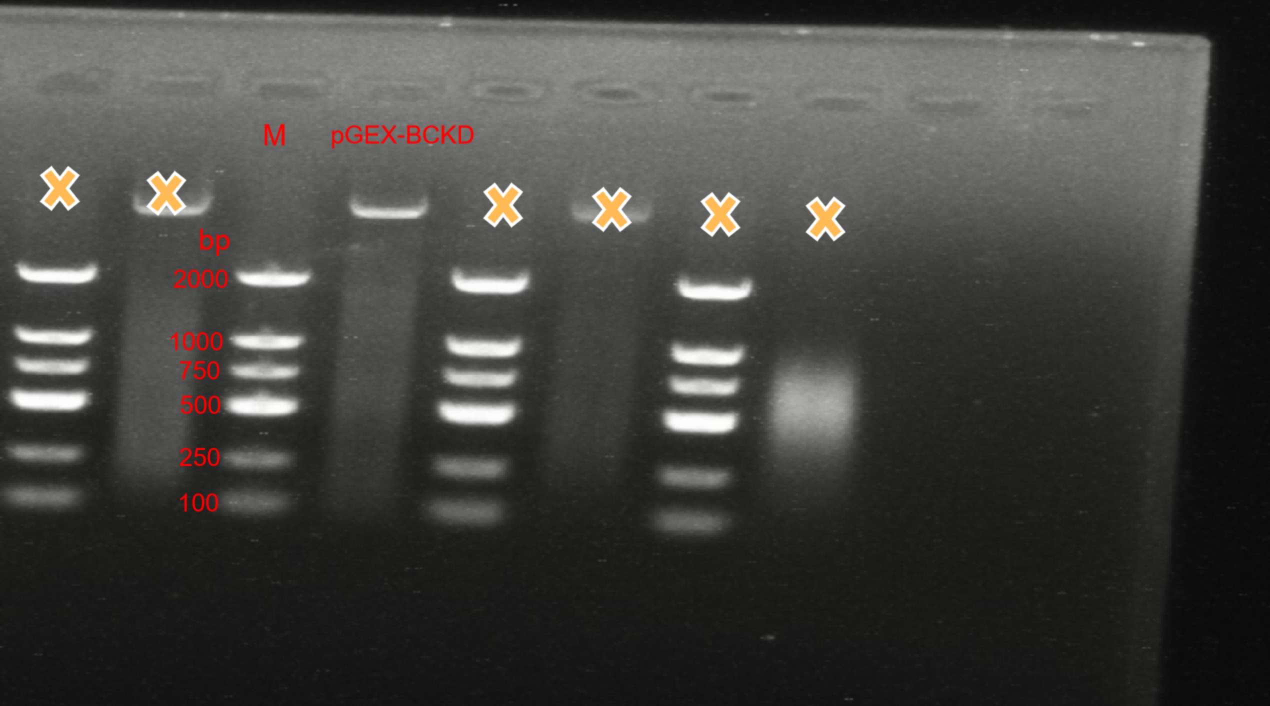

Figure 2 Agarose gel electrophoresis of pGEX-BCKD

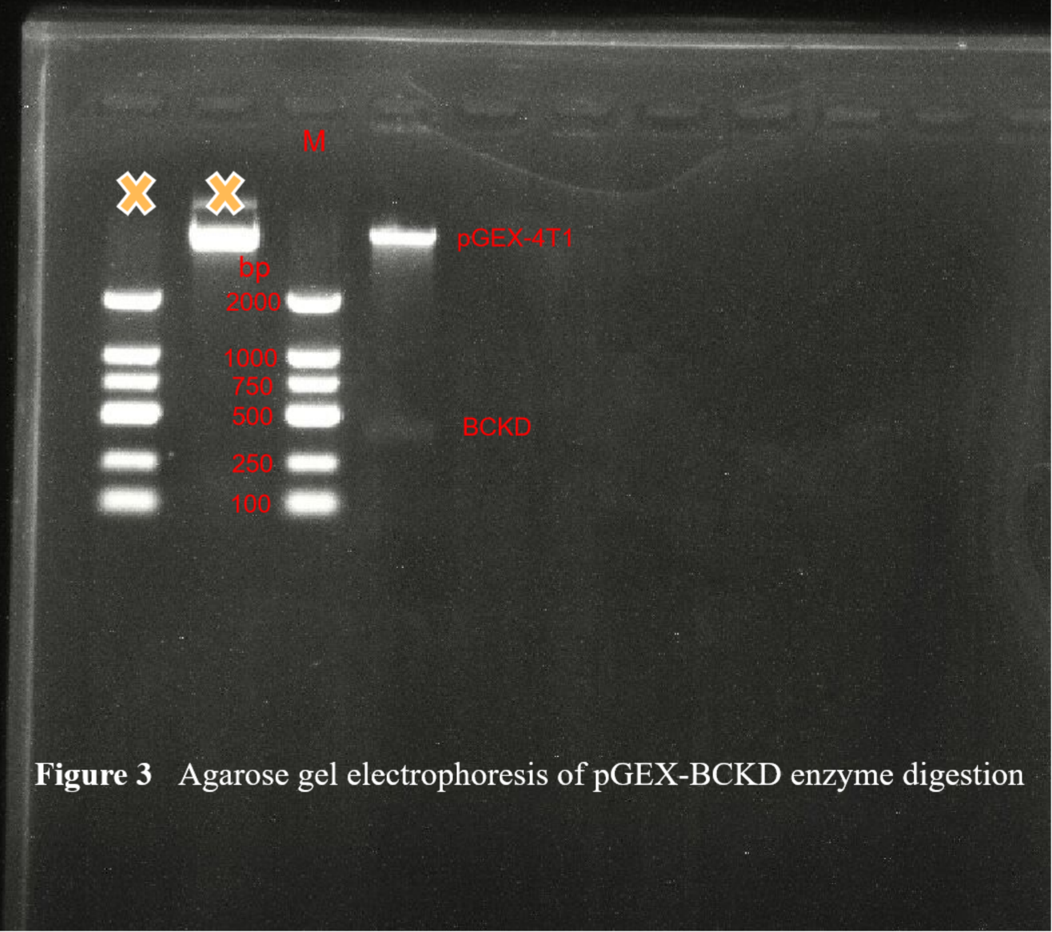

Figure 3 Agarose gel electrophoresis of pGEX-BCKD enzyme digestion

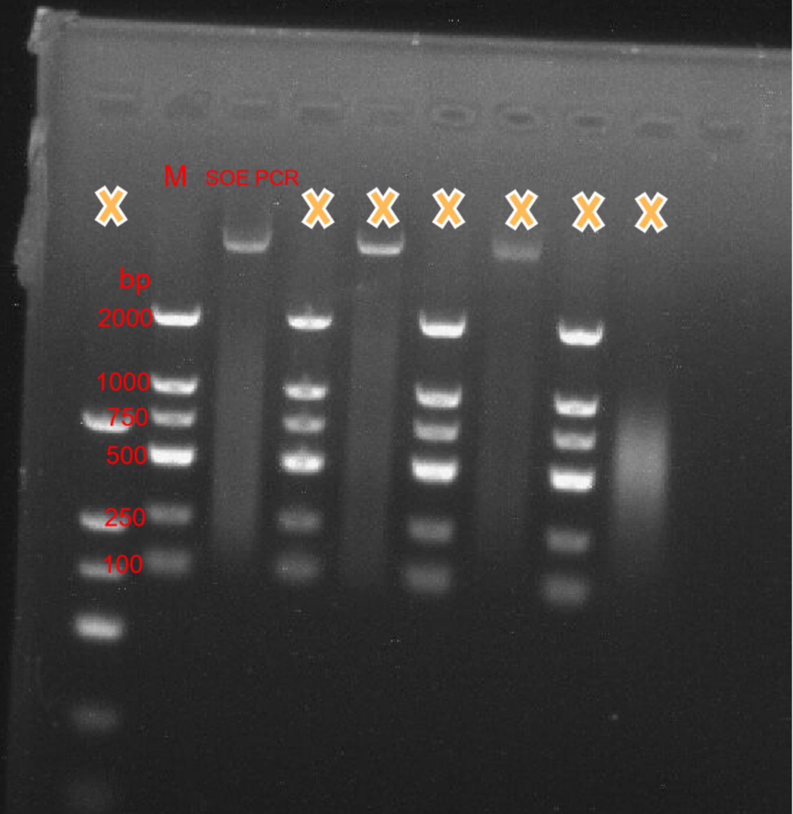

Figure 5 Agarose gel electrophoresis of colony SOE PCR
